# CircTMCC1 enhances radioresistance in esophageal squamous cell carcinoma by upregulating MYC via miR-186-3p sponging

**DOI:** 10.1007/s12672-026-04944-y

**Published:** 2026-04-08

**Authors:** Hongtai Shi, Huiwen Chang, Ye Hang, Kaiguo Sun, Xiaojin Hu, Bin Chen, Jianxiang Song, Zhan Shi

**Affiliations:** 1 Department of Radiation Oncology, Affiliated Hospital 6 of Nantong University, Yancheng Third People’s Hospital, Yancheng, China; 2 Department of Thoracic Surgery, Affiliated Hospital 6 of Nantong University, Yancheng Third People’s Hospital, Yancheng, China

**Keywords:** circTMCC1, ESCC, Radioresistance, MYC

## Abstract

The involvement of circRNA in the radiosensitivity of various malignancies has been well established; however, its specific regulatory function and underlying mechanisms in esophageal squamous cell carcinoma (ESCC) radiosensitivity remain largely unexplored. In this study, differentially expressed circRNAs were identified in ESCC tissues and cells exhibiting either radiosensitivity or resistance. Notably, circTMCC1 was found to be upregulated in radioresistant ESCC tissues and was predominantly localized in the cytoplasm. In both in vivo and in vitro models, silencing of circTMCC1 enhanced the radiosensitivity of ESCC cells. Mechanistic investigations further demonstrated that circTMCC1 acts as a molecular sponge for miR-186-3p, thereby promoting MYC expression and contributing to radioresistance in ESCC. This study identifies a novel role for the circTMCC1/miR-186-3p/MYC axis in regulating radioresistance in ESCC, suggesting the potential for new therapeutic strategies in ESCC radiotherapy.

**Supplementary Information:**

The online version contains supplementary material available at 10.1007/s12672-026-04944-y.

## Background

In 2020, global estimates projected 604,100 new cases of esophageal cancer and 544,076 related deaths, with approximately half originating from Asia [1]. Esophageal cancer primarily presents as squamous cell carcinoma (SCC) and adenocarcinoma, with esophageal squamous cell carcinoma (ESCC) being the predominant type in China. For patients with locally advanced esophageal cancer who are not candidates for curative resection, radical concurrent chemoradiotherapy remains the standard treatment [2]. However, substantial variability exists in both the radiotherapy response and prognosis among ESCC patients, indicating individual differences in radiosensitivity [3]. Thus, identifying the factors influencing ESCC radiosensitivity and exploring molecular biomarkers are critical for developing personalized treatment strategies.

In recent years, increasing evidence has revealed that circRNAs are crucial for nearly all biological processes associated with tumours, including initiation, progression, metastasis, and recurrence [4]. CircRNAs can block or mitigate the inhibitory effect of miRNAs on gene expression by competitively sponging miRNA binding sites, thereby increasing the expression of target genes. This mechanism is commonly referred to as the competing endogenous RNA (ceRNA) pathway [5]. Research has established a significant link between circRNAs and the radiosensitivity of various malignancies [6–8]. However, studies exploring its association with ESCC radiosensitivity remain limited. In this investigation, we analysed publicly available circRNA expression profiles (accession numbers PRJCA019651 and PRJCA019553). CircTMCC1, whose expression markedly differed, was selected for further study to elucidate its role in regulating ESCC radiosensitivity at the molecular level.

## Methods

### ESCC tissue collection and cell culture

The histological specimens analysed were obtained from ESCC patients who underwent radical concurrent chemoradiotherapy at Yancheng Third People’s Hospital prior to treatment. None had received any prior antitumour therapy. Patients were categorized as "radiotherapy-sensitive" (Complete Response + Partial Response, n = 10) or "radiotherapy-resistant" (Stable Disease + Progressive Disease, n = 10) based on the degree of tumor regression observed via imaging before and after the full course of radiotherapy. The pathological diagnoses were independently confirmed by two senior pathologists at the hospital. During specimen collection and pretreatment consultations, patients and their families were informed, and informed consent was obtained in compliance with the ethics committee's regulations. This study was approved by the Yancheng Third People’s Hospital Ethics Committee (approval number [2024] LSY No. 131).

All the cell lines (KYSE30, KYSE70, KYSE150, TE-1, and Het-1a) were obtained from the Cell Bank of the Chinese Academy of Sciences and authenticated by short tandem repeat (STR) profiling (Shanghai Biowing Biotechnology Co., Ltd.) within 6 months before the experiments were performed. The STR profiles matched the corresponding entries in the ATCC database (https://www.atcc.org/). Mycoplasma contamination was tested for monthly using a Mycoplasma Detection Kit (Beyotime, Cat. No. C0296), and all the cell lines were confirmed to be mycoplasma-free throughout the study. The cells were maintained in DMEM (high-glucose) supplemented with 10% foetal bovine serum. The cultures were incubated at 37 °C, 5% CO_2_, and 95% relative humidity.

### CircRNA acquisition from ESCC tissues and cells.

CircRNA data were retrieved from Sun’s study and are available from the National Genomics Data Center (https://www.cncb.ac.cn/) under accession numbers PRJCA019651 and PRJCA019553 [7]. Raw sequencing data were subjected to strict quality control: adapter-contaminated reads, low-quality reads (Q20 < 80%) and reads with N base content > 10% were removed to obtain high-quality clean reads, with all samples achieving Q30 ≥ 90%, clean read ratio ≥ 95% and genome mapping ratio ≥ 85%. CircRNAs were jointly identified with a threshold of back-spliced reads ≥ 2, and their expression levels were normalized to TPM (Transcripts Per Million) for subsequent differential expression analysis.Logarithmic-phase TE-1 were irradiated with Elekta 6MV X-rays (dose rate: 4 Gy/min; 1.5 cm silicone compensator under culture flasks) at 1 Gy initially. After medium replacement and culture, surviving cells were digested and passaged. Upon re-entering logarithmic phase, irradiation was repeated (1 Gy, then 2 Gy and 4 Gy, 3 times each) to a cumulative dose of 21 Gy, yielding radioresistant TE-1R cell lines.

### qRT‒PCR

Total RNA was extracted using TRIzol reagent, and the RNA quantity and concentration were determined with a NanoDrop2000 microspectrophotometer. cDNA was synthesized using the PrimeScript RT Reagent Kit (Takara), followed by RT‒qPCR with the SYBR Green Realmaster Mix Kit (Tiangen) and ABI ViiA 7 (Applied Biosystems B.V.). GAPDH was used as an internal control for circRNA and mRNA, while U6 was used for miRNA normalization. The expression levels of circRNAs, mRNAs, and miRNAs were quantified via the 2^−ΔΔCt^ method, with the primer sequences shown in Supplementary Table 1. The validation of GAPDH, U6 stability under irradiation or circRNA modulation was showed in Supplementary Fig. 1.

### FISH

KYSE150 cells (1 × 10^5^) were fixed in 4% paraformaldehyde for 10 min, followed by rinsing with PBS. PBS solution was then precooled with 0.5% Triton X-100 for 15 min (4 °C). A PE-labelled probe targeting circTMCC1 (Geneseed) was synthesized and used for hybridization. The cell nuclei were incubated with the probe in hybridization buffer (Geneseed, China) at 37 °C for 16 h, followed by DAPI staining. Images were obtained using a fluorescence microscope (Olympus CX53, Japan).

### RIP

The RNA enrichment level was assessed via qRT‒PCR with a Magna RIP kit (Millipore, Billerica, MA, USA) according to the manufacturer’s protocol, and AGO2 or control IgG was used as the antibody.

### RNase R treatment

RNA (2 μg) was incubated with 5 U/μg RNase R (R7092L; Beyotime, China) at 37 °C for 30 min. Subsequent analysis of circTMCC1 and TMCC1 mRNA expression was performed using SYBR Green qPCR.

### Nuclear‒cytoplasmic fractionation

Total RNA was extracted with TRIzol reagent (Takara, Dalian, China). Both fractions of KYSE150 cells were isolated using the PARIS™ Kit (Thermo Fisher, USA). β-actin served as a marker for the cytoplasmic fraction, while U6 was used to verify the nuclear fraction.

### Western blotting

Cell lysis and protein extraction were performed using RIPA buffer, followed by protein quantification via the BCA assay. Equal amounts of protein from each group were loaded for SDS‒PAGE analysis. The primary antibodies used included c-MYC (Abcam, Cat. No. ab32072; host: rabbit; clone: Y69; dilution: 1:1000; molecular weight: 54 kDa), γ-H2AX (Cell Signaling Technology, Cat. No. 9718S; host: rabbit; clone: 20E3; dilution: 1:1000; molecular weight: 15 kDa), and GAPDH (Cell Signaling Technology, Cat. No. 5174S; host: rabbit; clone: 14C10; dilution: 1:1000; molecular weight: 37 kDa). The secondary antibody was anti-rabbit HRP (Cell Signaling Technology, Cat. No. 7074S, host: goat, dilution: 1:5000). After overnight incubation at 4 °C and TBST washing, the membranes were incubated with the secondary antibody for 1 h and then subjected to washing again. ECL detection was used, followed by exposure, development, fixation, scanning, and image analysis for quantification of the results. The gray values of protein bands were quantified using ImageJ software, and the relative expression level of the target protein was calculated as the ratio of the gray value of the target protein to that of the internal reference GAPDH.

### Colony formation assay

All irradiation procedures were performed using a Varian TrueBeam linear accelerator (Varian Medical Systems, Palo Alto, CA, USA) equipped with 6 MV X-rays. The irradiation parameters were as follows: dose rate = 3 Gy/min, field size = 5 cm × 5 cm, and ambient temperature = 25 °C. Cells were irradiated (0, 2, 4, 6, and 8 Gy) in a monolayer adherent state (after seeding and attachment for 24 h) to mimic in vivo tumour growth patterns. Cells were then cultured for 10–14 days, and colonies containing ≥ 50 cells were counted (single cells or no proliferative microcolonies were excluded). Automatic counting was performed using the “Colony Counter” plugin of ImageJ software, followed by manual verification and correction to avoid subjective errors. Each treatment group and dose was tested in 3 independent replicates, and the data are presented as the means ± standard errors of the mean (SEMs).

### Dual luciferase reporter assay (DLRA)

Bioinformatic prediction identified the binding sites. Genes containing wild-type circTMCC1 (circTMCC1-WT) and those with mutated circTMCC1 binding sites (circTMCC1-MUT), along with MYC gene fragments (MYC-WT) and corresponding MYC fragments with mutated binding sites (MYC-MUT), were synthesized, cloned and inserted into the psiCHECK2 fluorescent vector to construct luciferase reporter plasmids (Generbiol) (Supplementary Fig. 2). These constructs were cotransfected with miR-NC and miR-186-3p mimics into KYSE150 cells. Luciferase activity was subsequently measured following the protocol provided by the DLRA kit. Firefly luciferase activity normalized to Renilla luciferase activity, expressed as Relative Luciferase Activity.

### Animal experiments

The experiments conformed to the protocols approved by the Care and Use Committee and the Ethics Committee of Shanghai Chengxi Biotechnology Co., Ltd. (CX052207031). This study was conducted in accordance with the ARRIVE guidelines. All subsequent mouse experiments were performed in accordance with the relevant guidelines and regulations. Each group consisted of six BALB/c nude mice (purchased from Shanghai Chengxi Biotechnology Co., Ltd.), which were randomized to a control group or a circTMCC1 downregulation group. Stable cell lines were established by silencing circTMCC1 in KYSE150 cells. Following the establishment of xenograft tumours in 6-week-old BALB/c nude, a 12 Gy dose was administered when the tumours reached approximately 200 mm^3^. Tumour volumes were measured and recorded every five days after irradiation using callipers. After 30 days, the mice were euthanized by inhalation of carbon dioxide in a euthanasia chamber.

### Statistical methods

Statistical analysis was performed with GraphPad Prism 9.0. The data are presented as the means ± SEMs. To assess between-group differences, a t test was used, while one-way ANOVA was applied for comparisons among multiple groups. *P* < 0.05 indicated statistical significance, whereas *P* ≥ 0.05 indicated no significant difference. All experiments were conducted in triplicate (n = 3).

## Results

**Fig. 1:**
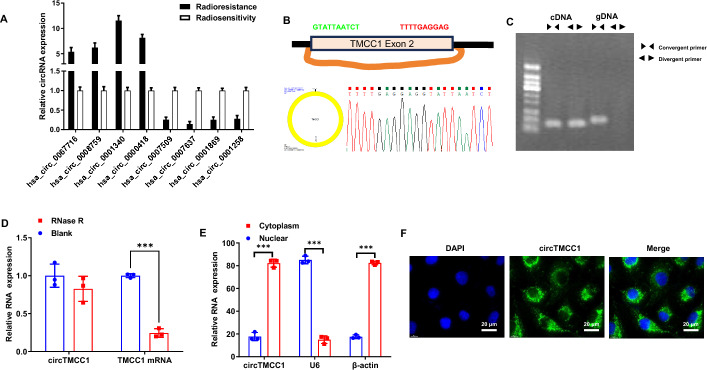
Circular characteristics of circTMCC1. **A** qRT‒PCR confirmed the differential expression of circRNAs in ESCC tissues exhibiting radiosensitivity and radioresistance. The error bars represent the means ± SEMs. **B** Sanger sequencing verified the circular nature of circTMCC1. **C** PCR amplification of cDNA and genomic DNA from circTMCC1 reverse transcription in KYSE150 cells. **D** RNase R treatment of KYSE150 cells demonstrated the stability of circTMCC1 relative to that of linear RNA. The error bars represent the means ± SEMs. **E** Nuclear‒cytoplasmic fractionation analysis was used to assess the subcellular localization of circTMCC1 in KYSE150 cells. The error bars represent the means ± SEMs. **F** FISH was used to visualize the subcellular distribution of circTMCC1 in KYSE150 cells. (***P < 0.001, **P < 0.01, *P < 0.05)

To study the role of circRNAs in ESCC radiosensitivity, this study integrated differential circRNA expression profiles from parental TE-1 and radiation-resistant TE-1R cell lines (as published by Sun et al.) with profiles from radioresistant and radiosensitive ESCC tissues [7]. On the basis of a fold change > 2 and *P* < 0.05, 2,102 circRNAs were identified as differentially expressed in cells, and 82 circRNAs were identified in tissues. The intersecting differentially expressed circRNAs were subsequently analysed, revealing 8 circRNAs (hsa_circ_0067716, hsa_circ_0008759, hsa_circ_0001340, hsa_circ_0000418, hsa_circ_0007509, hsa_circ_0007637, hsa_circ_0001869, and hsa_circ_0001258) based on consistent trends. These circRNAs were further validated by qRT‒PCR in ESCC tissues with varying degrees of radiosensitivity or radioresistance, with hsa_circ_0001340 showing the most significant differential expression (Fig. 1A). Hsa_circ_0001340 was subsequently selected for analysis.

The circBase database indicates that hsa_circ_0001340 is positioned at chr3:129599152-129599402 in the genome and is generated by the cyclization of exon 2 of the TMCC1 gene, which is 251 nt in length and is hereafter referred to as circTMCC1. PCR primers targeting the circTMCC1 cyclization site were designed and used for amplification, followed by Sanger sequencing of the product (Fig. 1B). In KYSE150 cells, PCR amplification of cDNA reverse-transcribed from circTMCC1 and gDNA revealed amplification of the circRNA fragment using divergent primers, while the corresponding gDNA fragment was not amplified, confirming the circular structure of circTMCC1 (Fig. 1C). After RNase R treatment, compared with linear RNA, circTMCC1 was more stable (Fig. 1D). Related assays demonstrated that circTMCC1 was predominantly localized in the cytoplasm of KYSE150 cells (Fig. 1E, F). The results confirm that circTMCC1 is a circular RNA that is stably expressed in the cytoplasm.

### circTMCC1 increased radioresistance in ESCC cells in vivo and in vitro

**Fig. 2:**
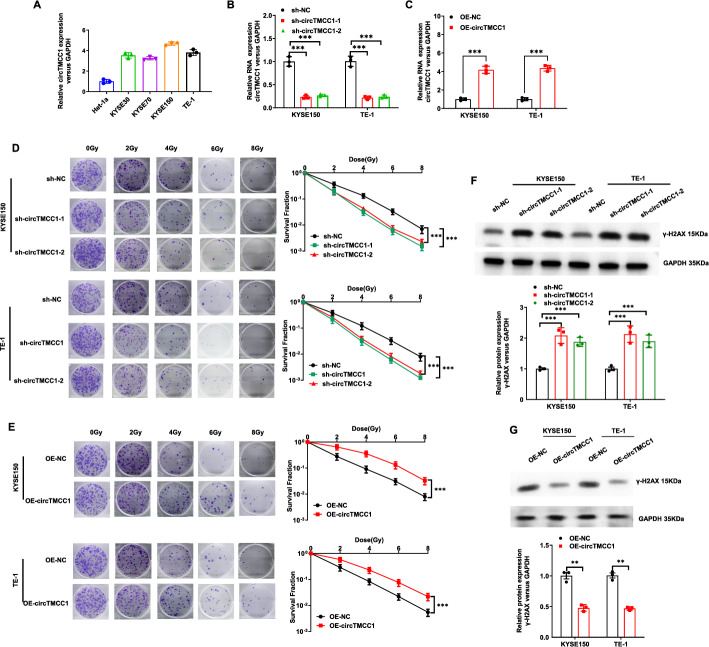
CircTMCC1 enhanced radioresistance in ESCC cells in vitro. **A** qRT‒PCR was used to quantify circTMCC1 expression in normal oesophageal epithelial cells (Het-1a) and ESCC cell lines (KYSE30, KYSE70, KYSE150, and TE-1). The error bars represent the means ± SEMs. **B** qRT‒PCR was used to assess the transfection efficiency following circTMCC1 knockdown in KYSE150 and TE-1 cells. The error bars represent the means ± SEMs. **C** qRT‒PCR was used to evaluate the transfection efficiency after circTMCC1 overexpression in KYSE150 and TE-1 cells. The error bars represent the means ± SEMs. **D** Colony formation assays were used to evaluate the survival of KYSE150 and TE-1 cells transfected with sh-NC or sh-circTMCC1 after 0, 2, 4, 6, and 8 Gy of radiation exposure. The error bars represent the means ± SEMs. **E** Colony formation assays were performed to assess the survival of KYSE150 and TE-1 cells transfected with OE-NC or OE-circTMCC1 after 0, 2, 4, 6, or 8 Gy irradiation. The error bars represent the means ± SEMs. **F** Western blot analysis of γ-H2AX expression in KYSE150 and TE-1 cells with circTMCC1 knockdown 6 h after -4 Gy irradiation. Densitometric quantification of protein bands was performed using ImageJ software, with relative γ-H2AX expression normalized to GAPDH (internal reference). All experiments were conducted with three biological replicates (n = 3). **G** Western blot analysis of γ-H2AX expression in KYSE150 and TE-1 cells with circTMCC1 overexpression 6 h after 4 Gy irradiation. Densitometric quantification of protein bands was performed using ImageJ software, with relative γ-H2AX expression normalized to GAPDH (internal reference). All experiments were conducted with three biological replicates (n = 3). (***P < 0.001, **P < 0.01, *P < 0.05)

CircTMCC1 expression in Het-1a and ESCC cell lines was assessed via qRT‒PCR, revealing higher levels in ESCC cells (Fig. 2A). To study the role of circTMCC1 in ESCC radiosensitivity, the cell lines with the highest circTMCC1 expression, KYSE150 and TE-1, were selected for further investigation. Stable cell lines with either circTMCC1 overexpression or knockdown were generated, and the expression levels were confirmed (Fig. 2B, C). Colony formation assays demonstrated that circTMCC1 overexpression in KYSE150 and TE-1 cells markedly increased cell survival, whereas circTMCC1 knockdown significantly reduced survival (Fig. 2D, E). Western blot analysis of γ-H2AX, a marker of the DNA damage response, was conducted 6 h after 4 Gy irradiation. The results revealed a substantial reduction in γ-H2AX expression in circTMCC1-overexpressing cells, but its expression was notably elevated in circTMCC1 knockdown cells (Fig. 2F, G). These findings collectively indicate that circTMCC1 contributes to increased radioresistance in ESCC cells.

**Fig. 3:**
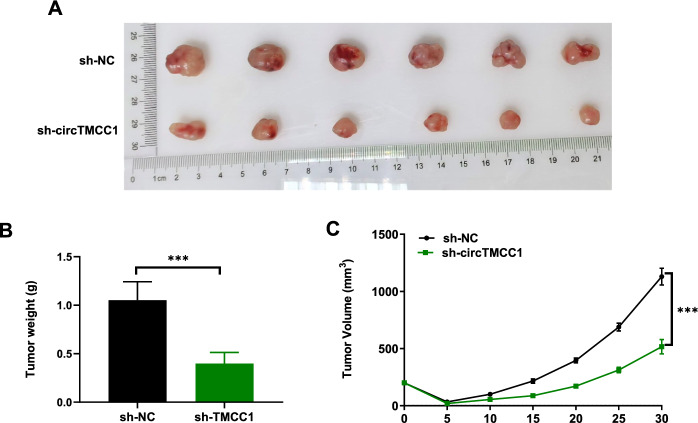
Downregulation of circTMCC1 expression enhanced the radiosensitivity of ESCC cells in vivo. **A** Representative images of excised tumours. **B** Tumour weights of subcutaneously implanted tumours in mice. The error bars represent the means ± SEMs. **C** Tumour growth curves in the subcutaneous model. The error bars represent the means ± SEMs. (***P < 0.001, **P < 0.01, *P < 0.05)

To examine the impact of circTMCC1 on tumour growth in vivo, tumour-bearing BALB/c nude mice were established using a KYSE150 cell line with stable downregulation of circTMCC1 and its control cells. When the tumour volume reached 200 mm^3^, the mice received a single 12 Gy dose of irradiation. The tumour volume was monitored every 5 days after irradiation. After 30 days, the mice were anaesthetized and sacrificed, and their tumours were excised (Fig. 3A). The circTMCC1 downregulation group exhibited reduced tumour weight, slower tumour growth, and smaller tumour volume (Fig. 3B, C). These results support the role of circTMCC1 in enhancing the radioresistance of ESCC cells in vivo.

### circTMCC1 could sponge miR-186-3p, thereby promoting MYC expression and causing radioresistance in ESCC

**Fig. 4:**
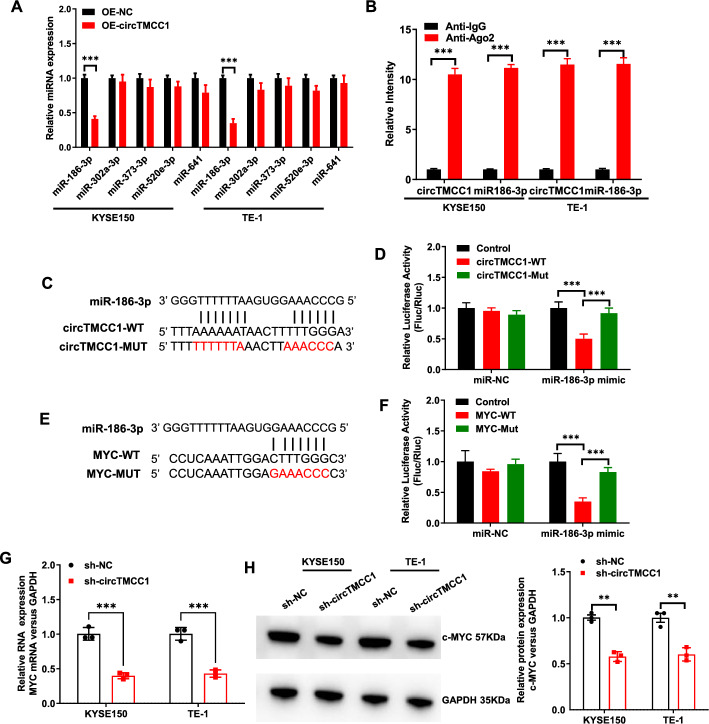
CircTMCC1/miR-186-3p/MYC interact as a ceRNA. **A** qRT‒PCR analysis of miR-186-3p, miR-302a-3p, miR-373-3p, miR-520e-3p, and miR-641 expression in KYSE150 and TE-1 cells following OE-NC or OE-circTMCC1 treatment. The error bars represent the means ± SEMs. **B** RIP analysis of circTMCC1 and miR-186-3p enrichment in KYSE150 and TE-1 cells. The error bars represent the means ± SEMs. **C** Complementary nucleotide sequences of circTMCC1-WT and miR-186-3p and the mutant sequence of circTMCC1-MUT. **D** Dual luciferase reporter assay: circTMCC1-WT and circTMCC1-MUT luciferase vectors were cotransfected with miR-186-3p into KYSE150 cells, after which luciferase activity was measured. The error bars represent the means ± SEMs. **E** Complementary nucleotide sequences of MYC-WT and miR-186-3p and the mutant sequences of MYC-MUT. **F** Dual luciferase reporter assay: MYC-WT and MYC-MUT luciferase vectors were cotransfected with miR-186-3p into KYSE150 cells, after which luciferase activity was measured. The error bars represent the means ± SEMs. **G** qRT‒PCR analysis of MYC mRNA expression in KYSE150 and TE-1 cells after sh-NC or sh-circTMCC1 treatment. The error bars represent the means ± SEMs. **H** Western blot analysis of c-MYC protein expression in KYSE150 and TE-1 cells after sh-NC or sh-circTMCC1 treatment. Densitometric quantification of protein bands was performed using ImageJ software, with relative c-MYC expression normalized to GAPDH (internal reference). All experiments were conducted with three biological replicates (n = 3). (***P < 0.001, **P < 0.01, *P < 0.05)

CircRNAs, which are typically localized in the cytoplasm, exert their biological functions primarily through a ceRNA mechanism [4]. Given that circTMCC1 is predominantly cytoplasmic, it is hypothesized to operate via this mechanism. To investigate the molecular pathway of circTMCC1, potential miRNA binding sites were predicted using circBank and miRDB. The top five predicted miRNAs from both databases were miR-186-3p, miR-302a-3p, miR-373-3p, miR-520e-3p, and miR-641. Subsequent qRT‒PCR validation revealed that miR-186-3p expression was downregulated in ESCC cells overexpressing circTMCC1 (Fig. 4A). RIP assays further confirmed that circTMCC1 and miR-186-3p were enriched in the Ago2 antibody group (Fig. 4B). To validate this interaction, a DLRA was conducted. circTMCC1-WT and circTMCC1-MUT luciferase plasmids were cotransfected with miR-186-3p into KYSE150 cells (Fig. 4C). The overexpression of miR-186-3p led to a marked decrease in luciferase activity in cells treated with the circTMCC1-WT vector but had no effect on cells transfected with the circTMCC1-MUT or empty vector (Fig. 4D). These results confirm that circTMCC1 directly binds to miR-186-3p, with the binding site located within the mutated region.

**Fig. 5:**
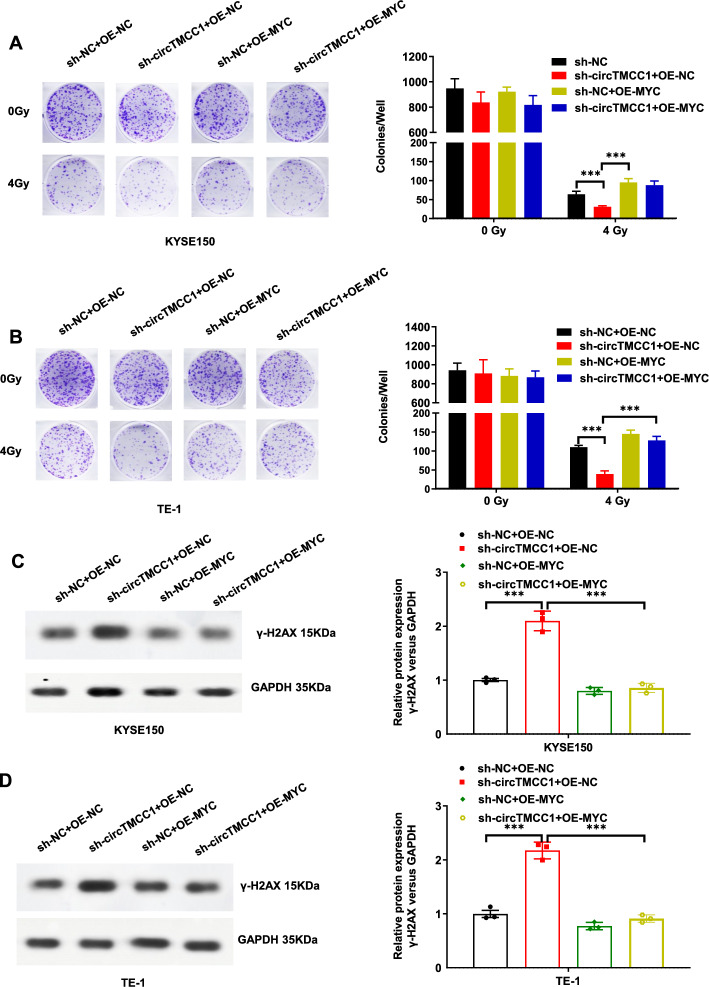
MYC overexpression reversed the increase in radiosensitivity induced by circTMCC1 downregulation. **A** Colony formation assays were performed to assess the survival of KYSE150 cells transfected with sh-NC + OE-NC, sh-circTMCC1 + OE-NC, sh-NC + OE-MYC, or sh-circTMCC1 + OE-MYC following 0 and 4 Gy of radiation exposure. The error bars represent the means ± SEMs. **B** Colony formation assays were conducted on TE-1 cells transfected with the aforementioned plasmid combinations after 0 and 4 Gy of radiation. The error bars represent the means ± SEMs. **C** Western blotting was used to measure γ-H2AX expression in KYSE150 cells transfected with sh-NC + OE-NC, sh-circTMCC1 + OE-NC, sh-NC + OE-MYC, or sh-circTMCC1 + OE-MYC following 4 Gy irradiation for 6 h. Densitometric quantification of protein bands was performed using ImageJ software, with relative c-MYC expression normalized to GAPDH (internal reference). All experiments were conducted with three biological replicates (n = 3). **D** Western blotting was also used to assess γ-H2AX expression in TE-1 cells transfected with the same plasmid combinations after 4 Gy irradiation for 6 h. Densitometric quantification of protein bands was performed using ImageJ software, with relative c-MYC expression normalized to GAPDH (internal reference). All experiments were conducted with three biological replicates (n = 3). (***P < 0.001, **P < 0.01, *P < 0.05)

Bioinformatics analysis (TargetScan and miRDB) and relevant literature have suggested that MYC may serve as a potential target of miR-186-3p [9]. Luo et al. demonstrated that LINC00460 acts as a ceRNA to sponge miR-186-3p, thereby upregulating MYC expression and promoting immune escape in colorectal cancer [9]. To test this hypothesis, MYC-WT and MYC-MUT luciferase plasmids were constructed (Fig. 4E) and cotransfected into KYSE150 cells with either the miR-186-3p mimic or the miR-NC. DLRA revealed that miR-186-3p overexpression decreased luciferase activity in the MYC-WT plasmid group but not in the empty vector or MYC-MUT plasmid groups (Fig. 4F). Thus, MYC was a target of miR-186-3p, whose binding site was a mutation site. Downregulation of circTMCC1 expression in KYSE150 and TE-1 ESCC cells led to decreases in MYC mRNA and protein levels (Fig. 4G, H), suggesting a potential ceRNA mechanism involving circTMCC1, miR-186-3p, and MYC. To further validate this hypothesis, KYSE150 and TE-1 cells were subjected to the following four transfection treatments: (1) sh-NC + OE-NC, (2) sh-circ TMCC1 + OE-NC, (3) sh-NC + OE-MYC, and (4) sh-circTMCC1 + OE-MYC. Colony formation assays demonstrated that circTMCC1 knockdown enhanced cellular radiosensitivity, whereas MYC overexpression reversed circTMCC1-induced radiosensitivity in both KYSE150 and TE-1 cells (Fig. 5A, B). Furthermore, γ-H2AX expression was assessed by Western blotting 6 h after irradiation, revealing that MYC overexpression significantly reduced γ-H2AX levels in circTMCC1 knockdown cells (Fig. 5C, D). These results collectively indicate that circTMCC1 is a molecular sponge for miR-186-3p, thereby upregulating MYC expression and contributing to radioresistance in ESCC.

## Discussion

ESCC represents a major malignancy of the digestive tract and poses a significant threat to human health. For patients with locally advanced ESCC for whom radical resection is not feasible, concurrent chemoradiotherapy remains the standard treatment approach. However, substantial variability in the response to radiotherapy persists among patients. These findings highlight the urgent need to investigate the mechanisms underlying resistance to radiotherapy and identify potential strategies to increase treatment sensitivity in patients with oesophageal cancer.

CircRNAs were initially regarded as transcriptional noise without significant biological function [10]. However, advancements in high-throughput sequencing have revealed their critical involvement in tumour biology [4, 11]. Furthermore, the role of circRNAs in modulating tumour radiosensitivity has been reported [6–8, 12–14]. In ESCC, Sun et al. demonstrated that m6A-modified circCREBBP interacts with IGF2BP3 to destabilize MYC, thereby enhancing radiosensitivity [7]. He et al. reported that circVRK1 diminished ESCC radiosensitivity via regulation of the miR-624-3p/PTEN axis and the PI3K/AKT signalling pathway [15]. Additionally, Ma et al. reported that downregulation of circPRKCI expression suppressed oesophageal cancer progression and enhanced radiosensitivity through modulation of the miR-186-5p/PARP9 axis [16]. In the current study, differentially expressed circRNAs associated with radiosensitivity and radioresistance in ESCC were identified using a public database and validated via qRT‒PCR. Among these, circTMCC1 was found to be overexpressed in radioresistant ESCC tissues. Colony formation assays and γ-H2AX detection confirmed that circTMCC1 promoted radioresistance in ESCC cells. Moreover, animal studies have demonstrated that downregulation of circTMCC1 expression increases the radiosensitivity of ESCC.

This study further investigated the molecular mechanism underlying the radioresistance mediated by circTMCC1 in ESCC. It was determined that circTMCC1 was predominantly localized in the cytoplasm, with the most likely regulatory mechanism being the ceRNA pathway. Epigenetic remodelling under oxidative stress drives tumour metastasis [17]. The role of miRNA interplay in cancer therapeutic interventions emphasizes that targeting miRNA-mediated regulatory networks can improve treatment efficacy [18]. Bioinformatics analysis revealed candidate miRNAs with binding sites complementary to those of circTMCC1, revealing that circTMCC1 negatively regulates miR-186-3p expression in ESCC cells. Subsequent RIP and DLRA analyses confirmed direct binding between circTMCC1 and miR-186-3p. MiR-186-3p has been established as a tumour suppressor in various malignancies [9, 19–24]. In colorectal cancer, LINC00460 can sponge miR-186-3p, thereby increasing the expression of MYC, CD47, and PD-L1, which promotes immune evasion [9]. Similarly, in gastric cancer, circUGGT2 sponges miR-186-3p to upregulate MAP3K9, promoting cell proliferation and increasing cisplatin resistance [19]. In cervical cancer, miR-186-3p targets and downregulates IGF1, thereby suppressing the PI3K-Akt signalling pathway and mitigating tumorigenesis [20]. These studies have established miR-186-3p as a tumour suppressor in various malignancies, with MYC, a key oncogene, identified as one of its target genes [9]. MYC is a classic oncogene that drives radioresistance in multiple malignancies by enhancing DNA damage repair and suppressing the accumulation of γ-H2AX [25]. In our study, the circTMCC1/miR-186-3p/MYC axis exerts its radioresistance-promoting effect via this classic regulatory pathway: circTMCC1 acts as a molecular sponge for the tumor-suppressive miR-186-3p to relieve its post-transcriptional inhibition of MYC, and the upregulated MYC subsequently reduces radiotherapy-induced γ-H2AX expression (Figs. 2F, G, 5C, D), thereby alleviating DSB damage in ESCC cells and enhancing radioresistance. Consistent with previous reports that miR-186-3p exerts radiosensitizing effects by targeting oncogenes in various tumors, our study further expands its functional target repertoire in ESCC by validating MYC as its novel downstream target mediating radioresistance. Moreover, the negative correlation between circTMCC1 and γ-H2AX expression, and the positive correlation between MYC and γ-H2AX expression in our experimental results, further confirm that this axis modulates ESCC radioresistance by regulating the core DNA damage repair pathway, which is highly consistent with the well-characterized molecular basis of tumor radioresistance. The results of the present study revealed that circTMCC1 is a sponge for miR-186-3p, thereby promoting MYC expression through this interaction. This finding established a mutual ceRNA relationship among the three genes, and rescue experiments demonstrated that MYC upregulation can reverse the radiosensitivity induced by circTMCC1 downregulation.

This study has several limitations that should be acknowledged. The present study included a small number of ESCC clinical samples (10 cases in the radiotherapy-sensitive group and 10 in the radioresistant group) from a single center, which may introduce selection bias. The results cannot be directly generalized to clinical practice, nor can a quantitative association be established between circTMCC1 expression and radiotherapy efficacy or prognosis in ESCC patients.circTMCC1 may be a candidate target for ESCC radiosensitization only after its correlation with radiotherapy efficacy and prognosis is further validated in multicenter, large-sample ESCC clinical cohorts.

Overall, the results of this study demonstrate that circTMCC1 increases MYC expression by sponging miR-186-3p, thereby activating the MYC pathway and promoting radioresistance in ESCC. This study identifies the circTMCC1/miR-186-3p/MYC axis as a potential regulatory pathway underlying ESCC radioresistance, which may serve as a promising candidate target for radiosensitization strategies in ESCC. However, its clinical applicability requires further validation in preclinical and clinical studies.

## Supplementary Information

Additional file 1.

Additional file 2.

## Data Availability

The datasets analysed during the current study are available from the National Genomics Data Center (https://www.cncb.ac.cn/) under accession numbers PRJCA019651 and PRJCA019553. Other data generated during and/or analysed during the current study are available from the corresponding author upon reasonable request.
