# Supplementary material for: CircTMCC1 enhances radioresistance in esophageal squamous cell carcinoma by upregulating MYC via miR-186-3p sponging
Source: Discov Oncol. 2026 Apr 8;17:771. doi: 10.1007/s12672-026-04944-y (PMC13199535; doi:10.1007/s12672-026-04944-y)
Supplement: Supplementary file 1 — Additional file 1. [file 12672_2026_4944_MOESM1_ESM.docx]

Supplementary Table 1 Sequences of primers used in the study

| Gene | primer sequence (5’-3’) |
| --- | --- |
| hsa_circ_0067716-Forward | TGCCTCCACCAGTAAAAGACA |
| hsa_circ_0067716-Reverse | AGCTGGACAGTCAAGATGGT |
| hsa_circ_0008759-Forward | TTCTCCATCAAAGGCCAAGG |
| hsa_circ_0008759-Reverse | CAATGAGCTCTGGAATGCCC |
| circTMCC1-Divergent Primer-Forward | TTACAACTGTTCATCCTCACCT |
| circTMCC1- Divergent Primer-Reverse | AAATCTTGTGACCAAAGGCAAC |
| circTMCC1-Convergent Primer-Forward | GCTGACGCTCGGTGAACAGT |
| circTMCC1-Convergent Primer-Reverse | TCCCAGCAATAGCTTCCCTTTCT |
| hsa_circ_0000418-Forward | ATTGGAGTAAATCAACCAAAAC |
| hsa_circ_0000418-Reverse | TAACTTTGTAAAGTGGTCTTTGTG |
| hsa_circ_0007509-Forward | ATGGCGGTTTCATGTGCAA |
| hsa_circ_0007509-Reverse | CAGGGGCGTCAACATTTCAT |
| hsa_circ_0007637-Forward | GTTTCCCCGCAAATGACTGG |
| hsa_circ_0007637-Reverse | GTTCCCACTGTTTAAAAGGCCT |
| hsa_circ_0001869-Forward | CAGTGGACCTCAAAATACATTGT |
| hsa_circ_0001869- Reverse | TCTGGGATCAATGGCGGAAT |
| hsa_circ_0001258-Forward | AGCTGCTGGACTTCCTGTGCA |
| hsa_circ_0001258-Reverse | TTCAGGCAAAACAGAAAAACA |
| miR-186-3p RT-primer | GTCGTATCCAGTGCAGGGTCCGAGGTA  TTCGCACTGGATACGACCCCAAA |
| miR-186-3p-Forward | GCCGAGGCCCAAAGGTGAATTT |
| miR-186-3p-Reverse | CAGTGCAGGGTCCGAGGTAT |
| MYC-Forward | GTCAAGAGGCGAACACACAAC |
| MYC-Reverse | TTGGACGGACAGGATGTATGC |
| GAPDH-Forward | ACAACTTTGGTATCGTGGAAGG |
| GAPDH-Reverse | GCCATCACGCCACAGTTTC |
| β-Actin-Forward | CATGTACGTTGCTATCCAGGC |
| β-Actin Reverse | CTCCTTAATGTCACGCACGAT |
| U6 RT-primer | CGCTTCACGAATTTGCGTGTCAT |
| U6 Forward | GCTTCGGCAGCACATATACTAAAAT |
