# Supplementary material for: CircTMCC1 enhances radioresistance in esophageal squamous cell carcinoma by upregulating MYC via miR-186-3p sponging
Source: Discov Oncol. 2026 Apr 8;17:771. doi: 10.1007/s12672-026-04944-y (PMC13199535; doi:10.1007/s12672-026-04944-y)
Supplement: Supplementary file 2 — Additional file 2. [file 12672_2026_4944_MOESM2_ESM.pdf]

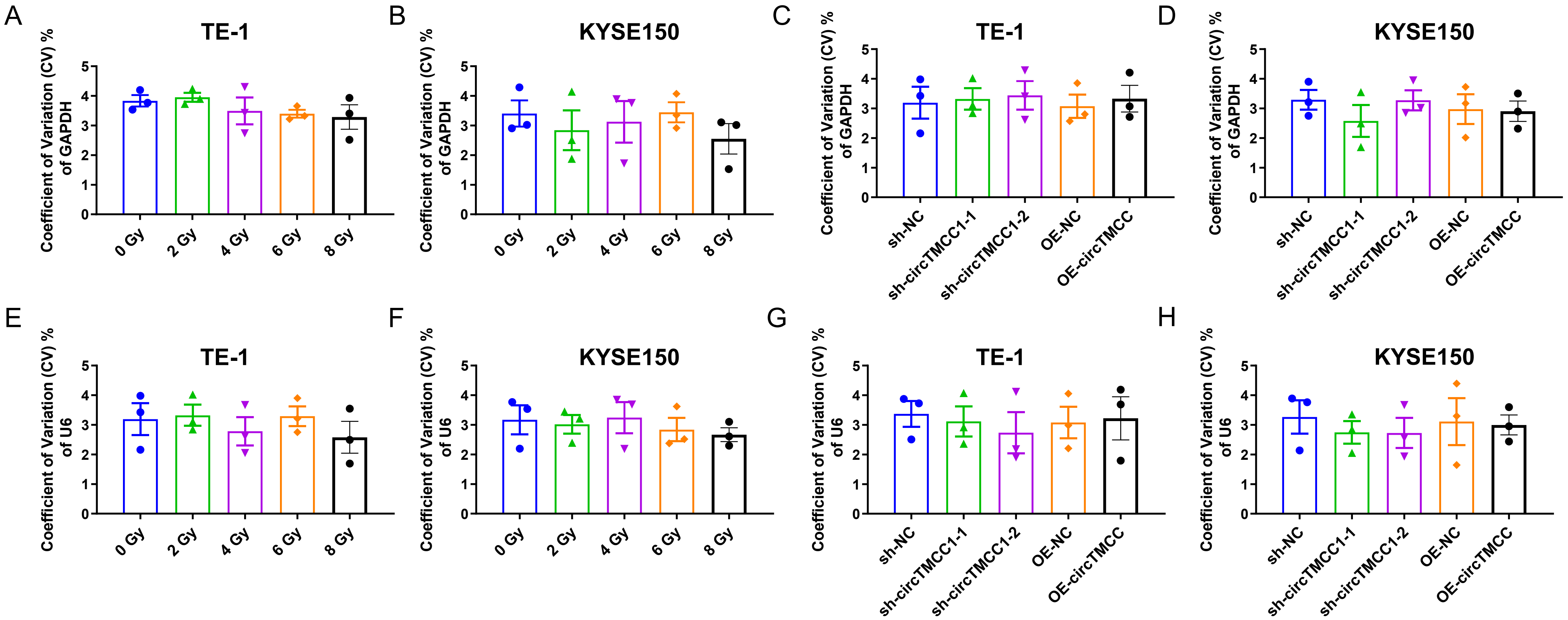

Figure 1: A-B. qRT-PCR results showed the coefficient of variation (CV) of GAPDH under irradiation (0, 2, 4, 6, 8 Gy) in TE-1 and KYSE150;
C-D. qRT-PCR results showed the coefficient of variation (CV) of GAPDH after circTMCC1 modulation in TE-1 and KYSE150;
E-F. qRT-PCR results showed the coefficient of variation (CV) of U6 under irradiation (0, 2, 4, 6, 8 Gy) in TE-1 and KYSE150;
G-H. qRT-PCR results showed the coefficient of variation (CV) of U6 after circTMCC1 modulation in TE-1 and KYSE150;

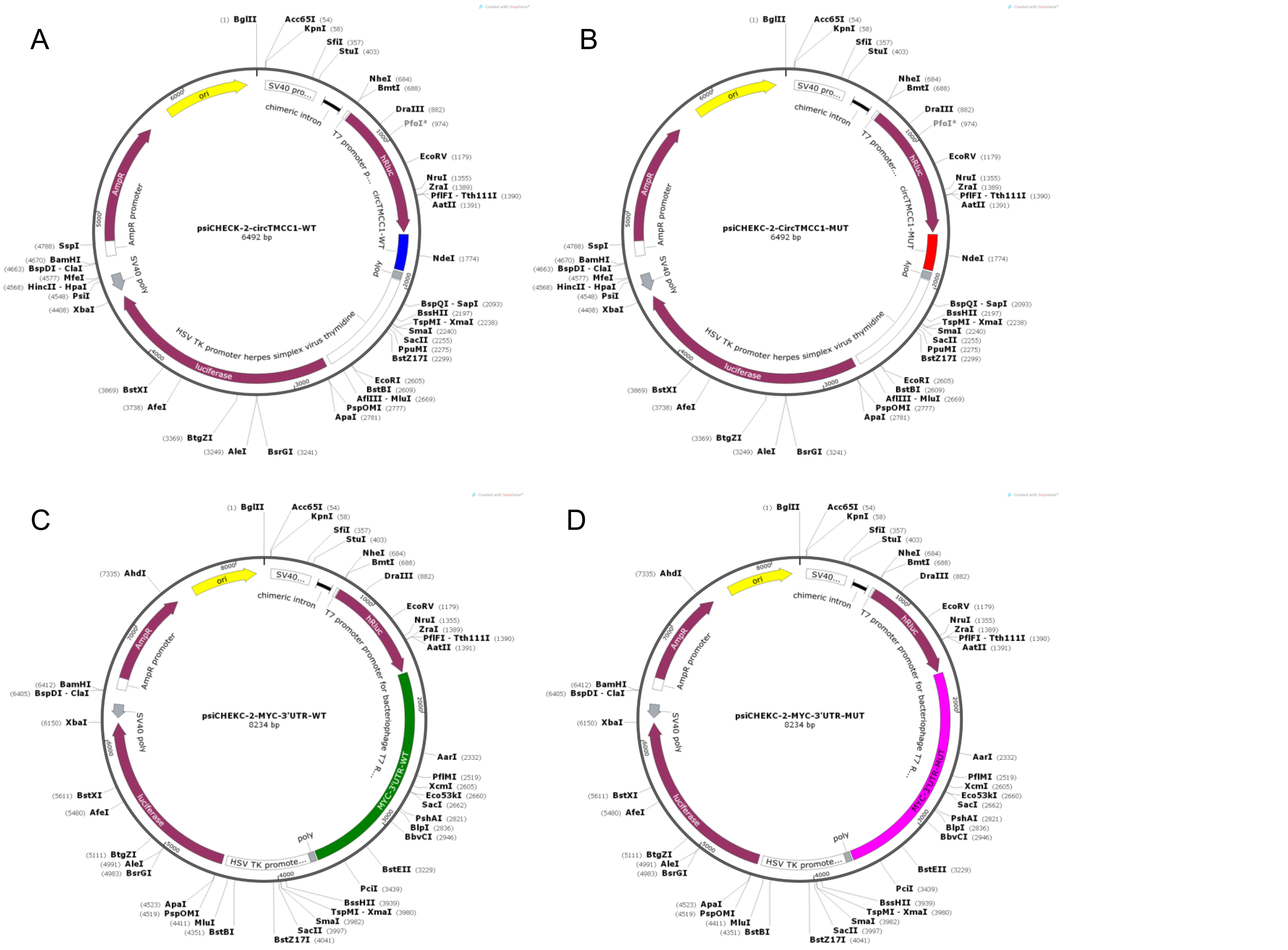

Figure 2: A. The vector maps of circTMCC1-WT plasmid. B. The vector maps of circTMCC1-MUT plasmid. C. The vector maps of MYC-WT plasmid. D. The vector maps of MYC-MUT plasmid.
